# Evaluation of a contactless neonatal physiological monitor in Nairobi, Kenya

**DOI:** 10.1136/archdischild-2021-322344

**Published:** 2021-11-05

**Authors:** Dee Wang, William M Macharia, Roseline Ochieng, Dorothy Chomba, Yifat S Hadida, Roman Karasik, Dustin Dunsmuir, Jesse Coleman, Guohai Zhou, Amy Sarah Ginsburg, J Mark Ansermino

**Affiliations:** 1 Department of Anesthesiology, Pharmacology and Therapeutics, The University of British Columbia, Vancouver, British Columbia, Canada; 2 Department of Pediatrics, Aga Khan University, Nairobi, Kenya; 3 EarlySense, Ramat Gan, Tel Aviv, Israel; 4 Centre for International Child Health, Vancouver, British Columbia, Canada; 5 Center for Clinical Investigation, Brigham and Women’s Hospital, Boston, Massachusetts, USA; 6 Clinical Trials Center, University of Washington, Seattle, Washington, USA

**Keywords:** technology, intensive care units, neonatal, neonatology

## Abstract

**Background:**

Globally, 2.5 million neonates died in 2018, accounting for 46% of under-5 deaths. Multiparameter continuous physiological monitoring (MCPM) of neonates allows for early detection and treatment of life-threatening health problems. However, neonatal monitoring technology is largely unavailable in low-resource settings.

**Methods:**

In four evaluation rounds, we prospectively compared the accuracy of the EarlySense under-mattress device to the Masimo Rad-97 pulse CO-oximeter with capnography reference device for heart rate (HR) and respiratory rate (RR) measurements in neonates in Kenya. EarlySense algorithm optimisations were made between evaluation rounds. In each evaluation round, we compared 200 randomly selected epochs of data using Bland-Altman plots and generated Clarke error grids with zones of 20% to aid in clinical interpretation.

**Results:**

Between 9 July 2019 and 8 January 2020, we collected 280 hours of MCPM data from 76 enrolled neonates. At the final evaluation round, the EarlySense MCPM device demonstrated a bias of −0.8 beats/minute for HR and 1.6 breaths/minute for RR, and normalised spread between the 95% upper and lower limits of agreement of 6.2% for HR and 27.3% for RR. Agreement between the two MCPM devices met the a priori–defined threshold of 30%. The Clarke error grids showed that all observations for HR and 197/200 for RR were within a 20% difference.

**Conclusion:**

Our research indicates that there is acceptable agreement between the EarlySense and Masimo MCPM devices in the context of large within-subject variability; however, further studies establishing cost-effectiveness and clinical effectiveness are needed before large-scale implementation of the EarlySense MCPM device in neonates.

**Trial registration number:**

NCT03920761.

**What is already known on this topic?**

- Sustainable Development Goal 3’s focus on treatment and prevention of acute illness calls for development and optimisation of innovations for neonatal care in low-resource settings.
- Continuous monitoring of neonatal heart and respiratory rates is rarely performed in low-resource settings due to poor availability of monitoring technologies and high acquisition costs.
- Almost all studies of automated monitoring technologies have been conducted in high-resource settings, making it difficult to assess these technologies for use in low-resource settings.

**What this study adds?**

- This is a novel evaluation of the accuracy of a contactless multiparameter continuous monitoring device compared with a reference standard in neonates in a low-resource setting.
- We describe a method for and some of the challenges in performing monitoring device accuracy studies in neonates.
- Our study indicates acceptable agreement between the EarlySense device and the Masimo Rad-97 reference device for measurement of heart and respiratory rates.

## Introduction

High neonatal mortality rates persist in Sub-Saharan Africa, with 27 deaths/1000 live births, a rate 10 times higher than rates in high-resource settings.^1^ Interventions are urgently needed, particularly in low-resource African settings, if Sustainable Development Goal Target 3.2 of reducing neonatal mortality to 12 deaths/1000 live births is to be achieved by 2030.^2^

Major causes of neonatal mortality include complications of preterm birth, asphyxia, and infectious diseases which are preventable and treatable.^3^ Technologies that can detect vulnerability and prompt interventions have great potential to improve neonatal survival. Multiparameter continuous physiological monitoring (MCPM) devices could be key in identifying high-risk neonates. Studies have demonstrated that monitoring of vital sign trends can predict clinical deterioration and allow for timely intervention and improvement in health outcomes.^4–8^ However, neonatal MCPM devices are not available in most low-resource settings due to barriers such as the high cost of equipment and a lack of health personnel with appropriate training.^9 10^

A better understanding of if and how MCPM devices can be adapted and optimised for use in neonates in low-resource settings is critical. Our study’s primary outcomes were agreement of heart rate (HR) and respiratory rate (RR) measurements comparing the EarlySense (EarlySense, Israel) investigational device to the Masimo Rad-97 pulse CO-oximeter with capnography (Masimo Corporation, USA) reference device. We hypothesised that the EarlySense device would agree within a priori*–*defined thresholds for HR and RR measurements when compared with the reference device and show minimal bias.

## Methods

### Study design and procedures

We conducted an iterative prospective study at Aga Khan University, Nairobi (AKU-N), a tertiary healthcare facility in Kenya, to assess agreement of HR and RR measurements from the EarlySense investigational device with those from the Masimo Rad-97 reference device.^10^ The Masimo Rad-97 device was selected based on its capability for high-resolution data collection and neonatal capnometry and pulse oximetry. EarlySense’s contactless wireless piezoelectric sensor can be placed under a patient’s mattress to detect ballistic vibrations from respiratory chest wall movement and cardioballistic movements from ejection of blood (online supplemental appendix A).

10.1136/archdischild-2021-322344.supp1 Supplementary data

During Masimo Rad-97 reference device verification, we tested functionality and estimated within-neonate and between-neonate variability to pre-specify thresholds for agreement comparisons.^11^ We completed an initial round of open-label data collection from both devices in which complete reference device data were shared with EarlySense prior to analysis. Three rounds of closed-label analysis followed, in which the first 15–30 min of reference device data from each neonate were provided to EarlySense to confirm time synchronicity across data sources (online supplemental appendices B, C). All reference device data were provided to EarlySense on completion of each subsequent round of data analysis to optimise HR and RR detection algorithms.

**Figure 1:**
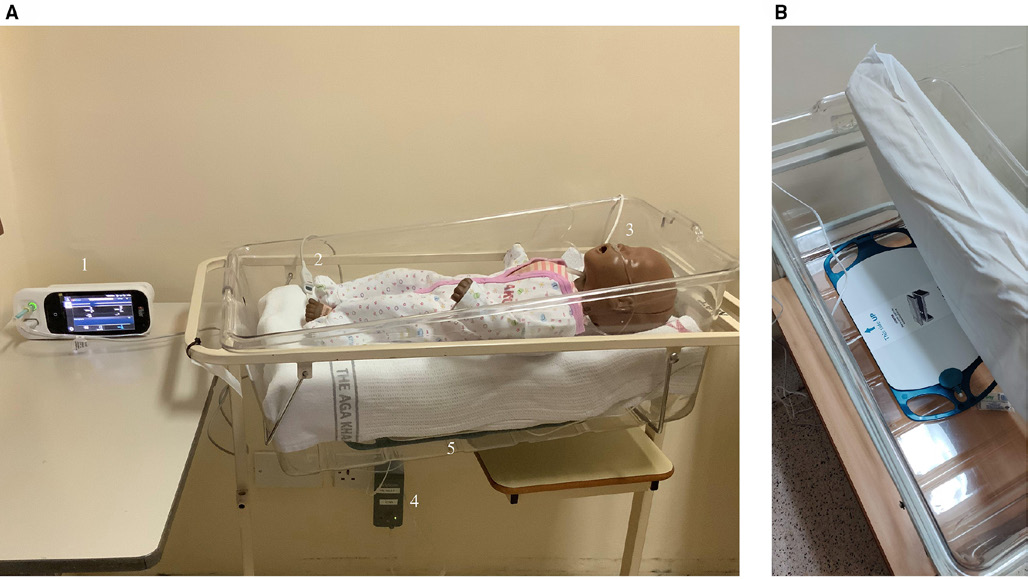
(A) Overview of the set-up showing the Masimo Rad-97 device with touchscreen interface (1), pulse oximeter probe (2), Nomoline for capnography (3), EarlySense processing unit (4), EarlySense under-mattress sensor (5). (B) Close-up of the EarlySense sensor under a mattress. The EarlySense sensor is connected to the processing unit that processes, stores data and sends results wirelessly to the remote display unit where the data are presented.

Caregivers of neonates delivered at AKU-N during the study period were approached for recruitment into the study and trained study clinicians obtained informed consent prior to determining eligibility for enrolment (table 1). Enrolled neonates were simultaneously monitored by both devices (figure 1; online supplemental video 1).

10.1136/archdischild-2021-322344.supp2 Supplementary video

**Table 1:** Eligibility criteria and study definitions

Eligibility criteria	
Inclusion criteria	Corrected age of <28 days Caregivers were willing and able to provide informed consent and were available for follow-up for the duration of the study
Exclusion criteria	Receiving continuous positive airway pressure or mechanical ventilation Skin abnormalities in the nasopharynx and/or oropharynx Contraindication to skin sensor application Known arrhythmia Congenital abnormality requiring major surgical intervention Any medical or psychosocial condition or circumstance that would interfere with study conduct or for which study participation could put the neonate’s health at risk
**Study definitions**	
Epoch	A 60 s period of time
Breath	One cycle of neonate-initiated inhalation and exhalation
Breath start	End of a waveform trough (low point) where the carbon dioxide level starts to ascend
Respiratory rate (RR) manual counting protocol	A breath was counted if the waveform peak reached either 15 mmHg or the average peak of the epoch, AND the waveform trough dipped below the average trough of the epoch plus 10 mmHg Each plot was counted by two independent readers and averaged If the difference in the counts was >5, a third independent reader counted the plot If the third count was within 5 breaths of either previous count, the average of the two closest counts was used
RR epoch exclusion criteria	RR epoch excluded if (i) the difference between the epoch count and median RR was >10, (ii) either value was <15, (iii) the capnogram contained a digital artefact or (iv) if there was lack of inter-reader manual count agreement
RR median calculation	For each breath in an epoch, an instantaneous RR could be calculated by the breath duration, and the median of all these instantaneous RR values in an epoch was then calculated
Heart rate (HR) median calculation	Heart beats were identified by the timing of the Masimo Rad-97 reference device plethysmograph quality index (PO-SQI) which occur at the peak of each heart beat. For each heart beat in an epoch, an instantaneous HR could be calculated by the time between the previous heart beat peak and current heart beat peak, and the median of all these instantaneous HR values in an epoch was then calculated
Adequate signal quality	EarlySense device: A signal quality score ≥0.7 for at least 50% of the epoch Masimo Rad-97 reference device: plethysmograph quality index (PO-SQI) threshold >150 for 100% of the epoch for HR, and capnography quality score (CO_2_-SQI) threshold ≥2 for at least 90% of the epoch for RR

### Data processing and selection

To ensure accurate, reliable and consistent data collection, we conducted the study in accordance with the Guideline for Good Clinical Practice/International Standards Organization (ISO) 14 155.

We randomly selected epochs of HR and RR with sufficient signal quality for each neonate recording (table 1). The first 5 min and last minute of recorded data, and first minute prior to and 5 min following any device or sensor disconnection were excluded to remove potentially poor-quality data typically present during sensor placement and removal. For the open-label round, we selected 20 epochs from ten different neonates. For each closed-label round, we selected ten epochs from 20 different neonates. No overlapping epochs were selected, and missing data were excluded from analyses. Median HR and RR values were calculated for the selected epochs (table 1). Manual RR counting was performed for each epoch using capnograms (online supplemental appendix D). Any RR epochs meeting exclusion criteria were replaced with another randomly selected epoch (table 1). See online supplemental appendix E for more details.

### Statistical analysis

To quantify the agreement between the EarlySense and reference devices, the normalised bias (95% CI) and the normalised spread between the 95% limits of agreement (LOA) were calculated by dividing the bias and spread between the 95% LOA by the overall reference HR or RR mean value.^12^ Based on a verification phase, the acceptable *a priori*-defined normalised spread between the 95% upper and lower LOAs of 30% was selected for both RR and HR.^11^ We calculated the root-mean-square deviation (RMSD) for each comparison. Clarke error grids were constructed with zones of 20% discrepancy to improve clinical interpretability of results.

## Results

We enrolled 94 neonates from 9 July 2019 to 8 January 2020, 76 of which were included for analysis (online supplemental appendix F). Three neonates withdrew prior to the minimum data collection time, and 15 neonates were excluded due to issues with data identification (incorrect date/times recorded) or time synchronisation (10), insufficient (less than 1 hour) length of recording (4) or poor data quality (1). There were six recordings where only one of the vital signs was usable for analysis due to poor data quality. There were no adverse events due to monitoring.

Equal numbers of female and male neonates from the neonatal high dependency unit (64.5%), postnatal and maternal wards (34.2%), and neonatal intensive care unit (1.3%) were included for analysis. Median gestational age was 38 (range 28–42) weeks and primary diagnoses were healthy post-delivery (35.5%), prematurity (27.6%), jaundice (17.1%), hypoglycaemic (6.6%), respiratory distress syndrome (5.3%), low birth weight (3.9%) and other (3.9%). We collected 280 hours of data (online supplemental appendix G), with a median recording length of 4 hours (range 73–423 min). See online supplemental appendix H for additional results.

**Figure 2:**
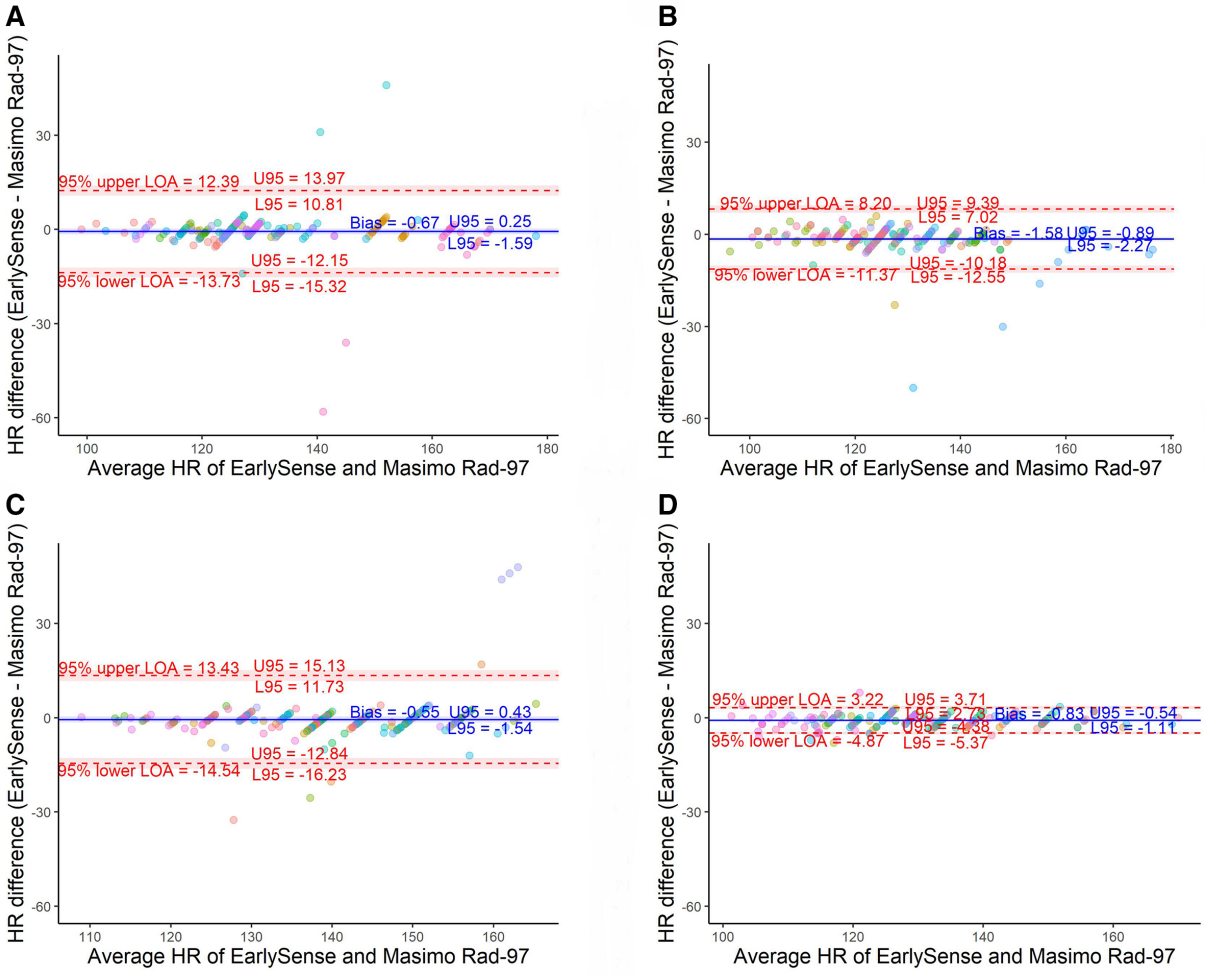
Bland-Altman plots for heart rate (HR). (A) Open-label round. (B) Closed-label round one. (C) Closed-label round two. (D) Closed-label round three. Colours indicate which participant neonate is associated with the measurement pair.

**Figure 3:**
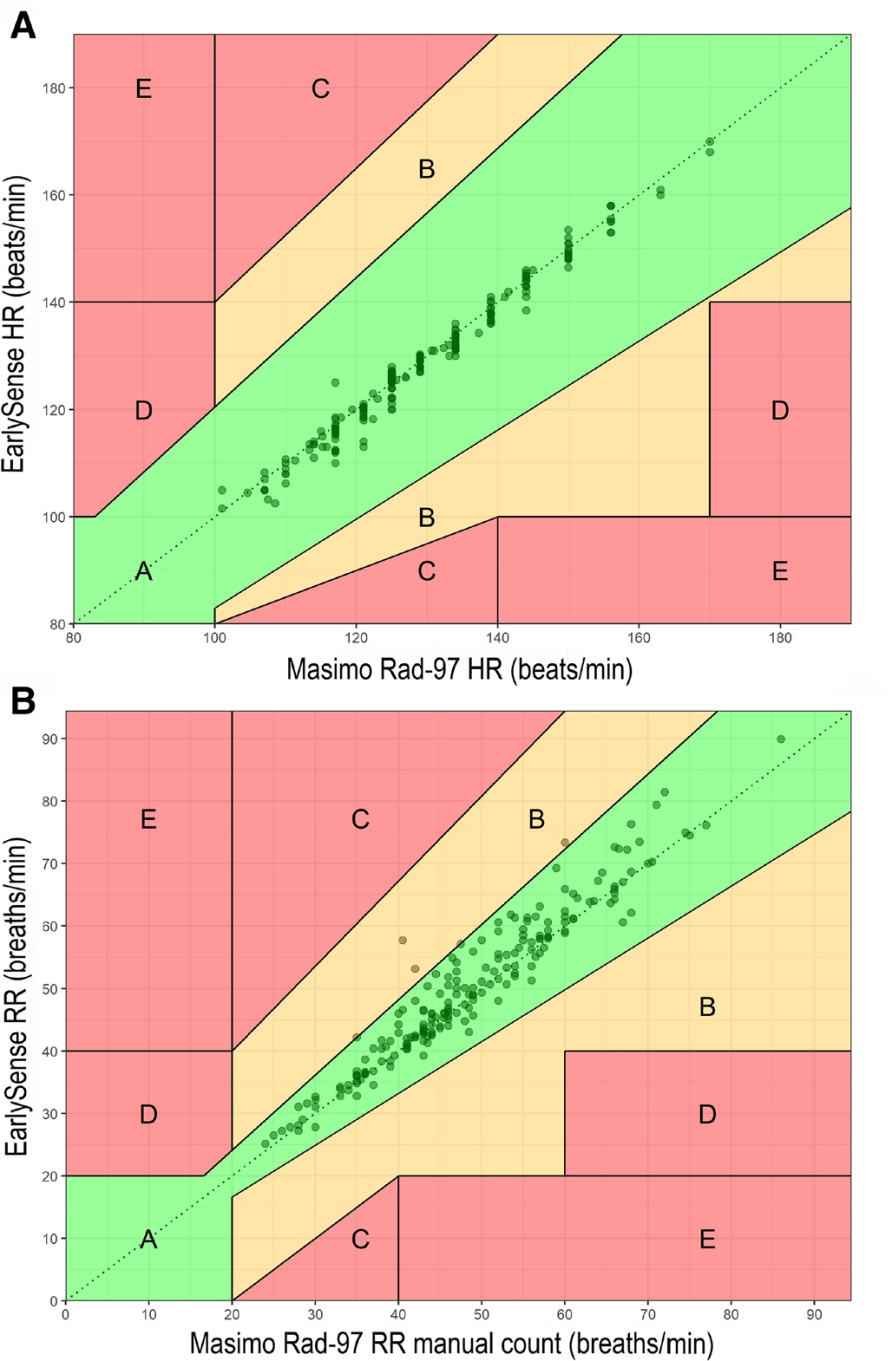
Clarke error grids for closed-label round three measurements. (A) Comparison of heart rate (HR) measurements. (B) Comparison of EarlySense respiratory rate (RR) to Masimo Rad-97 RR manual count. Each dot represents a data pair, with the colour intensity proportional to density of data pairs. Region A (in green) contains data pairs that are within 20% of the Masimo Rad-97 device value. Region B (in yellow) contains data pairs not within 20% that would not lead to unnecessary treatment. Regions C, D and E are in red. C includes data pairs leading to unnecessary treatment. D includes data pairs with a failure in detecting low or high HR/RR events and E includes data pairs where low and high HR/RR events are confused.

HR analysis identified a small negative average bias (range −1.58 to −0.55 beats/minute) with normalised spread of LOA meeting the a priori–defined threshold for all rounds (table 2; figure 2). A marked decrease in normalised spread between 95% LOA between closed-label rounds 2 and 3 (20.1 vs 6.2%) was observed. All EarlySense HR measurements were within 20% of the reference device values (figure 3).

**Table 2:** Results from Bland-Altman analysis for EarlySense investigational device versus Masimo Rad-97 reference device heart rate (HR) and respiratory rate (RR) measurements

	Open label	Closed-label round 1	Closed-label round 2	Closed-label round 3
**EarlySense HR compared with Masimo Rad-97 HR**				
Mean Masimo Rad-97 HR	132.3	128.2	139.1	130.2
Normalised bias (95% CI)	−0.5% (−1.2% to 0.2%)	−1.2% (−1.8% to −0.7%)	−0.4% (−1.1% to 0.3%)	−0.6% (−0.9% to −0.4%)
Normalised spread between 95% LOA (upper and lower 95% LOA)	19.7% (−10.4% to 9.4%)	15.3% (−8.9% to 6.4%)	20.1% (−10.5% to 9.7%)	6.2% (−3.7% to 2.5%)
Normalised RMSD	5.0%	4.1%	5.1%	1.7%
**EarlySense RR compared with Masimo Rad-97 RR manual count**				
Mean Masimo Rad-97 RR	48.0	51.7	54.2	48.5
Normalised bias (95% CI)	3.0% (1.4% to 4.6%)	0.3% (−1.0% to 1.7%)	2.5% (1.6% to 3.4%)	3.3% (2.4% to 4.3%)
Normalised spread between 95% LOA (upper and lower 95% LOA)	45.3% (−19.7% to 25.6%)	37.6% (−18.4% to 19.1%)	24.8% (−9.9% to 14.9%)	27.3% (−10.4% to 17.0%)
Normalised RMSD	11.9%	9.6%	6.8%	7.7%
**EarlySense RR compared with Masimo Rad-97 RR median**				
Mean Masimo Rad-97 RR	50.3	53.5	56.4	50.7
Normalised bias (95% CI)	−1.9% (−3.4% to −0.4%)	−3.1% (−4.4% to −1.8%)	−1.5% (−2.2% to −0.8%)	−1.2% (−1.9% to − 0.5%)
Normalised spread between 95% LOA (upper and lower 95% LOA)	41.6% (−22.7% to 18.9%)	35.9% (−21.1% to 14.9%)	19.2% (−11.1% to 8.2%)	19.8% (−11.1% to 8.7%)
Normalised RMSD	10.8%	9.7%	5.1%	5.2%

LOA, limits of agreement; RMSD, root-mean-square deviation.

**Figure 4:**
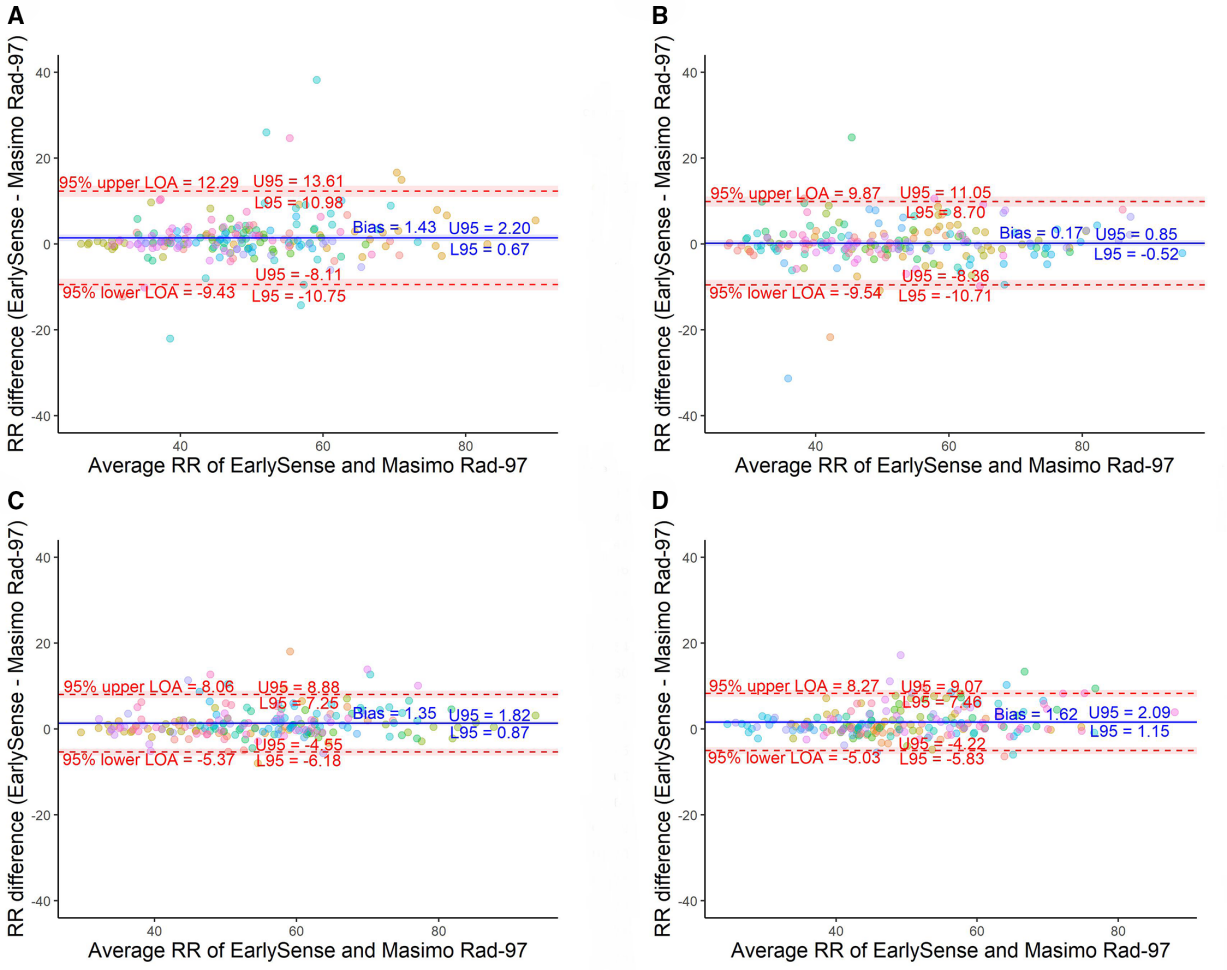
Bland-Altman plots for manual counts of respiratory rate (RR). (A) Open-label round. (B) Closed-label round one. (C) Closed-label round two. (D) Closed-label round three. Colours indicate which participant neonate is associated with the measurement pair.

RR analyses showed a small positive average bias in the EarlySense device (range 0.17–1.62 breaths/minute) when compared with reference device RR manual counts and a small negative average bias (range −1.65 to −0.63 breaths/minute) when compared with RR median values (table 2; figure 4). Normalised spread between 95% LOA decreased between the open-label and first two closed-label rounds, with results from closed-label rounds 2 and 3 meeting the a priori–defined threshold for both RR manual counts and median values. A marked decrease in normalised spread between 95% LOA from closed-label rounds 1 and 2 (37.6 vs 24.8% for RR manual counts; 35.9 vs 19.2% for RR medians) was observed. Absolute and normalised spreads of 95% LOA for RR median values were consistently smaller than RR manual counts when comparing the EarlySense and reference devices. Of the data pairs from closed-label round 3, only three (1.5%) EarlySense RR values were outside 20% of the reference device values (figure 3).

## Discussion

We found acceptable agreement between the EarlySense and reference devices for measurement of HR and RR in neonates by the final round of analysis, as evidenced by normalised spread between 95% LOA values below the a priori–defined threshold of 30%. The EarlySense algorithms were optimised between closed-label rounds 1 and 2 for RR, and between closed-label rounds 2 and 3 for HR. Upon optimisation, a marked narrowing of normalised spread between 95% LOA and reduction in bias was observed. Clarke error grids showed that all observations for HR and 197/200 for RR fell in region A (20% difference) (figure 3).

Selection of a reference standard is key when evaluating accuracy of a MCPM device. While ECG is the accepted reference standard for HR measurement, plethysmogram-derived HR, as used by the Masimo Rad-97 device, has been shown to be accurate even for comparisons of HR variability that require precise timing in identification of the heartbeat.^13^ The EarlySense device uses ballistocardiography (BCG). In comparison with ECG, heartbeats are less pronounced and more difficult to detect in BCG signals due to a lower signal-to-noise ratio^14^; yet, BCG signals have been shown to accurately estimate HR in adults.^15^

We selected capnography as the RR reference method.^16^ Agreement between RR measurements was impacted by within-neonate RR variability, which is particularly pronounced in neonates due to immature control of respiration, and normal and abnormal physiological responses such as periodic breathing and periods of apnoea.^17^ There are currently no recommended ISO standards for RR accuracy, though United Nations International Child’s Fund has suggested a maximum variability of ±2 breaths/minute in their acute respiratory infection diagnostic aid target product profile.^18^ This threshold may be unreasonably conservative when considering a neonate breathing at 60 breaths/minute, where a within-neonate variation of 2 breaths/minute would equate to a 3.3% variation. In setting the 30% threshold for the spread between 95% LOA, we considered the large within-neonate RR variability present in the verification phase data and avoided setting the target agreement threshold to be more restrictive than the within-neonate variation.^11^ We chose to normalise the spread between 95% LOA and used a percentage error threshold to allow for comparison of performance over a wide range of RR values. Use of a ±30% error threshold for LOA to determine whether to accept a new method in cardiac output method comparison studies was proposed in 1999 and has since been used extensively in the field.^19^

We compared EarlySense RR measurements to reference device RR manual counts and medians. Since clinical measurement of RR is typically determined by counting the number of breaths over a 1 min period, comparison of EarlySense RR values to RR manual counts was used as the primary comparison. However, manual count methods have several limitations. Capnograms can contain several different breathing patterns, such as periodic breathing and small-amplitude breaths, and it is unclear which breathing patterns should be included in a manual count and which should cause a minute to be excluded. In addition, rounding is a significant consequence of counting breaths. The rounding error may be as much as 6.7% if considering a RR of 15 breaths/minute and a rounding error of 1 breath/minute. We used a comparison between EarlySense RR and reference device RR median values since median values are more likely to reflect the underlying control of breathing. Medians are more robust than means, especially in the presence of extreme outliers, and are often used in automated device signal processing methods. This may explain the greater agreement when comparing the EarlySense RR to the reference device medians versus RR manual counts.

We reported RMSD values, as the accuracy of HR and RR has typically been reported as a percentage error rate (such as ±2%) compared with a reference standard.^20^ While RMSD is provided as a standardised metric when performing comparisons, RMSD does not consider the uncertainty in the reference method. In this evaluation, the normalised RMSD was 1.7% for HR, and 7.7% and 5.2% for RR manual counts and median values, respectively, in the final round (table 2). This was approximately 25% of the value of normalised spread between 95% LOA.

The study results indicate that the EarlySense MCPM device performs well when used to measure HR and RR as compared with the more invasive Masimo Rad-97 reference device. Advantages of the EarlySense device are that it is contactless and does not require disposable components. However, limitations include lack of MCPM when the neonate is not in bed, and lack of oxygen saturation monitoring. Notably, the Masimo Rad-97 capnography cannula was removed when the neonate was not in bed.

The accuracy of the EarlySense device was confirmed in a study population of high-risk adult surgical patients and in a small feasibility series with a paediatric and adult patient population in a sleep laboratory and intensive care unit setting.^21 22^ Other studies of monitoring technologies have been conducted in low-resource settings^23–26^; however, research conducted in neonates has largely been limited to studies in high-resource settings. Well-established methods for HR and RR monitoring exist, but limitations including inaccuracy, invasiveness, time-consuming application and high cost have prompted development of novel technologies.^27–29^ Novel contactless technologies are attractive as they avoid discomfort and risk of skin irritation. However, they have limitations of their own including motion artefacts, poor sensor coupling, poor-quality recordings and dependency on adequate lighting.^28 29^ There is a strong need for additional studies evaluating neonatal monitoring technology in low-resource settings.

Major strengths of our study were the independence of the investigators conducting the analysis, robust measurement of RR with high-resolution capnography, manual counting of breaths and randomised selection of minutes for comparison. However, our study site was better resourced than is typical in the region, and it is possible that the device may not have performed as well in a less-resourced environment. Currently, we are completing our next phase at Pumwani Maternity Hospital in Nairobi, Kenya, the largest referral maternal hospital in sub-Saharan Africa and a site more representative of a low-resource setting.

A substantial percentage (19.1%) of neonate recordings were not included for analysis due to data synchronisation and data identification issues (incorrect dates assigned). To minimise uncertainty in the reference device data and to avoid the EarlySense device missing the a priori threshold due to poor quality reference device data, we removed a substantial proportion of reference minutes from analysis and only included minutes with the highest quality reference data. In addition, we did not study clinical correlations or outcomes and were unable to assess the degree to which findings represented clinically significant differences. In the next phase of our study, we will assess the clinical feasibility of the EarlySense device with regard to accuracy, up-time, and event detection of high and low HR and RR events. Many of the neonates in this study were healthy, or relatively healthy, so it is possible that the agreement we observed may not reflect the sensor performance in critically ill neonates with higher or more irregular HRs and/or RRs. The EarlySense investigational device algorithms were developed in a cohort of neonates who were not critically ill to ensure that the initial evaluation and optimisation was performed in a more controlled environment, as is typically done for regulatory approval, before extending to more challenging cases and environments. We found the performance of this innovative non-invasive MCPM device to be promising in neonates. In a qualitative study, we found that healthcare administrators, healthcare providers and caregivers considered the EarlySense device to be feasible, useable and acceptable.^30^

Moving forward, it will be valuable to evaluate the EarlySense device for agreement with a reference device in neonates in intensive or critical care. A future study assessing the agreement of the EarlySense device HR and RR to ECG-derived HR and RR would strengthen the evidence for adoption of this novel contactless technology. In addition, evaluating the threshold and adaptive alerts provided by the device, and establishing cost-effectiveness, and clinical effectiveness will be necessary before large-scale implementation can be considered.

## Data Availability

Data are available in a public, open access repository. Data will be made available on completion of the secondary analyses of the data. We have ethics approval to deposit the data in an open access repository upon completion of the study.
