# Supplementary material for: Evaluation of a contactless neonatal physiological monitor in Nairobi, Kenya
Source: Arch Dis Child. 2021 Nov 5;107(6):558–64. doi: 10.1136/archdischild-2021-322344 (PMC9125375; doi:10.1136/archdischild-2021-322344)
Supplement: Supplementary data [file archdischild-2021-322344supp001.pdf]

APPENDICES

Appendix A

EarlySense Device Information

The thickness of the mattress does not affect accuracy of the Earlysense device, and a thicker mattress distributes the signal over a larger surface area such that the EarlySense device becomes less dependent on the sensor position under the mattress. The anticipated commercial acquisition cost of the EarlySense device is approximately $150 USD.

Wang D, *et al. Arch Dis Child* 2022; 107:558–564. doi: 10.1136/archdischild-2021-322344

Appendix B

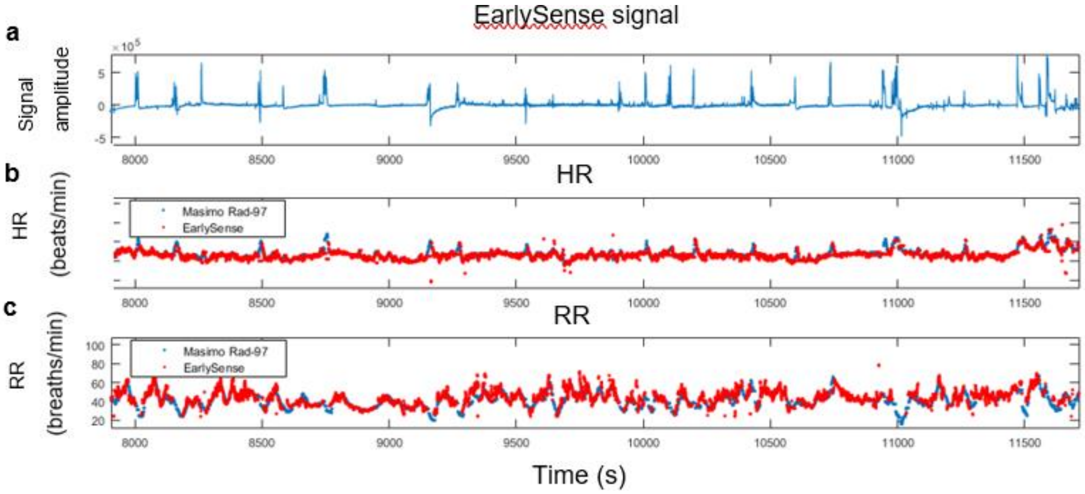

Figure shows time synchronization of signals from EarlySense and Masimo Rad-97 devices over a 1-hour period. **a** EarlySense raw data signal. **b** EarlySense (in red) and Masimo Rad-97 (in blue) heart rate (HR) detections synchronized. **c** EarlySense and Masimo Rad-97 respiratory rate (RR) detections synchronized.

Appendix C

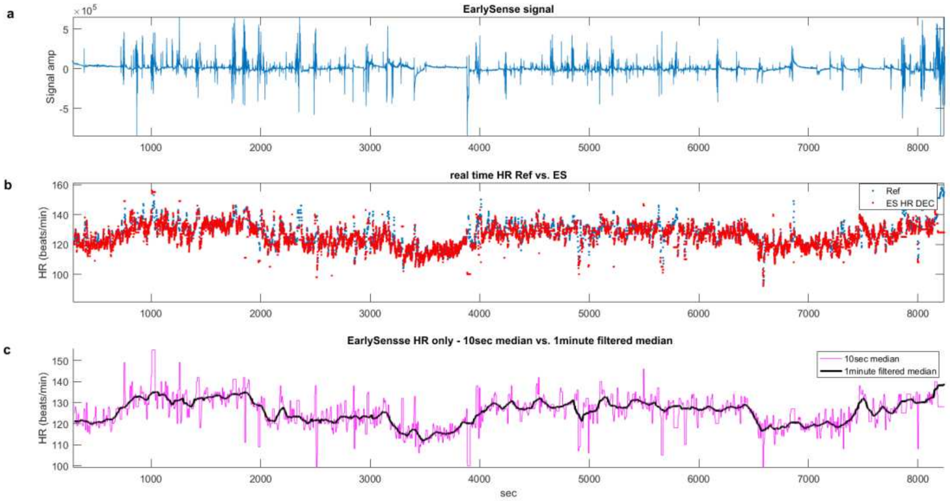

Figure shows time synchronization of signals from EarlySense and Masimo Rad-97 over a 2h:15min period. **a** EarlySense raw data signal. **b** EarlySense (in red) and Masimo Rad-97 (in blue) heart rate (HR) detections synchronized. **c**. EarlySense HR detections using 10 second median (in pink) and 1 minute filtered median (in black). Real-time measurement of HR is dependent on a 6 second signal and may suffer from artifacts. Anomaly detections are filtered out by only taking HR processed data between the 10th and 90th percentiles. A median value is calculated over the filtered data. If this median value and the median value from the last 10 seconds (non-filtered) crosses a threshold, an alert is raised.

Appendix D

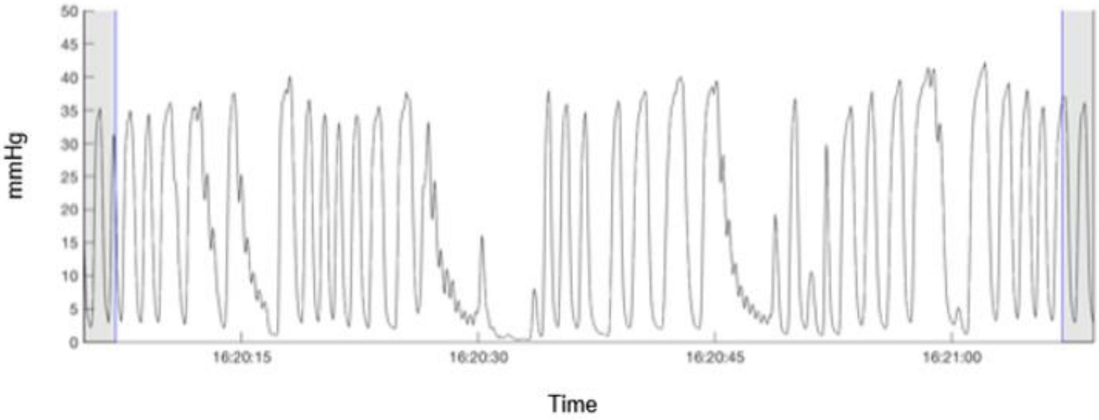

Figure shows Masimo Rad-97 60-second capnogram demonstrating typical irregularity of respiratory rate. The monitoring was conducted during a quiet period without external stimuli.

Wang D, *et al. Arch Dis Child* 2022; 107:558–564. doi: 10.1136/archdischild-2021-322344

Appendix E

Data processing and selection

We retrieved raw data collected in real-time from the reference device with a custom Android application. Data was parsed in C (Dennis Ritchie & Bell Labs, USA) to obtain plethysmograph waveform and plethysmograph quality index (PO-SQI) data at 62.5 hertz (Hz), and capnography (carbon dioxide (CO_2)) waveform data at approximately 20 Hz. We analyzed CO_2 waveform data using a breath detection algorithm developed in MATLAB (Math Works, USA) based on adaptive pulse segmentation. We developed a custom algorithm based on capnography features to determine the capnography quality index (CO_2-SQI).

The HR and RR from the EarlySense device were calculated every second from the output signal which was sampled at 240.5 Hz, using neonatal algorithms adapted from previously validated algorithms for adults.[21] Signal quality was determined for each measurement to assist with data analysis.

A sample size of 200 randomly selected epochs of data was used to ensure a narrow 95% confidence interval of +/- 0.24 standard deviation of between-device-measurement differences around the limits of agreement (LOA).

Wang D, *et al. Arch Dis Child* 2022; 107:558–564. doi: 10.1136/archdischild-2021-322344

Appendix F

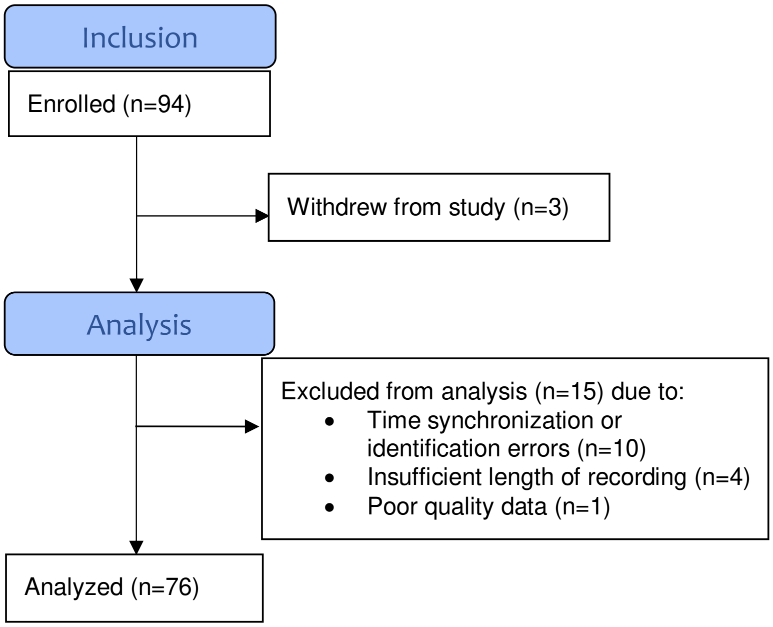

Figure of flow diagram showing enrolled neonatal participants.

Appendix G

Table showing overview of Masimo Rad-97 reference and EarlySense investigational device data from study neonates for the open-label (test) round and subsequent closed-label rounds

	Open-label round	Closed-label round one	Closed-label round two	Closed-label round three
Neonates included	11	20	22	23
Epochs per device (Masimo Rad-97/EarlySense)	2588/2297	5105/4018	4958/4187	4268/3898
Epochs meeting signal quality criteria (Masimo Rad-97/EarlySense)				
Heart rate	966/1449	1617/3026	1767/3192	1415/2354
Respiratory rate	984/1440	1683/2220	895/2751	1353/2319

Note: Total EarlySense device monitoring duration includes in-bed minutes only.

Wang D, *et al. Arch Dis Child* 2022; 107:558–564. doi: 10.1136/archdischild-2021-322344

Appendix H

Additional results

Of the recordings that were included for analysis, there was on average 21% of data missing from the EarlySense device, and 25% of HR and 30% of RR data missing from the Masimo Rad-97 device. Reasons for missing EarlySense data included the neonate being out of bed or sensor disconnections due to power outages. Reasons for missing Masimo data included the capnography and pulse oximetry probes being removed when the neonate was out of bed and sensor disconnections due to power outages. The Masimo Rad-97 capnography nasal cannula at times needed to be removed due to the fragility of these neonates, some of whom did not tolerate the nasal cannula. Power outages also contributed to missing data.

In a retrospective review of all of the data, we were unable to detect any significant episodes of apnea (> 20 seconds of no respiration associated with bradycardia).
